# Epidemiological and clinical characteristics of healthcare-associated infection in elderly patients in a large Chinese tertiary hospital: a 3-year surveillance study

**DOI:** 10.1186/s12879-020-4840-3

**Published:** 2020-02-10

**Authors:** Xia Zhao, Lihong Wang, Nan Wei, Jingli Zhang, Wenhui Ma, Huijie Zhao, Xu Han

**Affiliations:** 1 Hospital Infection Management Division, Xuanwu Hospital, Capital Medical University, No.45 Changchun Street, Xicheng District, Beijing, 100053 China; 2 School of Health Management and Education, Capital Medical University, Beijing, China

**Keywords:** Healthcare associated-infection, Elderly, Ventilator-associated pneumonia, Catheter-associated urinary tract infection, Central line-associated bloodstream infections, Pathogens

## Abstract

**Background:**

We analyzed the results of a 3-year surveillance study on the epidemiological and clinical characteristics of healthcare associated-infections (HAIs) in elderly inpatients in a large tertiary hospital in China.

**Methods:**

Real-time surveillance was performed from January 1, 2015 to December 31, 2017. All HAIs were identified by infection control practitioners and doctors. Inpatient data were collected with an automatic surveillance system.

**Results:**

A total of 134,637 inpatients including 60,332 (44.8%) elderly ≥60 years were included. The overall incidence of HAI was 2.0%. The incidence of HAI in elderly patients was significantly higher than that in non-elderly patients (2.6% vs. 1.5%, χ^2^ = 202.421, *P* < 0.01) and increased with age. The top five sites of HAIs in the elderly were the lower respiratory tract, urinary tract, blood stream, antibiotic-associated diarrhea, and surgical site. The five most common pathogens detected in elderly HAI patients were *Candida albicans*, *Klebsiella pneumonia*, *Acinetobacter baumannii*, *Escherichia coli*, and *Pseudomonas aeruginosa*. The incidence of ventilator-associated pneumonia in the elderly was lower than in the non-elderly, catheter-associated urinary tract infections were more common in elderly patients, and the rate of central line-associated bloodstream infection was similar between groups. The numbers of male patients and patients with comorbidities and special medical procedures (e.g., intensive care unit admission, cerebrovascular disease, brain neoplasms, hypertension, hyperlipidemia, diabetes mellitus, coronary artery disease, chronic obstructive pulmonary disease, malignant tumor, malignant hematonosis, and osteoarthropathy) were significantly higher in the elderly group, but the number of patients who underwent surgery was lower.

**Conclusion:**

We observed a significantly higher overall incidence of HAI in elderly inpatients ≥60 compared to non-elderly inpatients < 60 years, but the trend was different for device-associated HAIs, which was attributed to the higher rates of comorbidities and special medical procedures in the elderly group. The main HAI sites in elderly inpatients were the lower respiratory tract, urinary tract, and bloodstream, and the main pathogens were gram-negative bacilli and *Candida albicans*.

## Background

Modern societies have continually growing numbers of elderly inpatients. In 2017, there were an estimated 962 million people aged ≥60 years in the world, comprising 13% of the global population [1]. The population aged ≥60 is growing at an annual rate of ~ 3%. European countries currently have the greatest percentage of population aged ≥60 (25%). Rapid ageing will also soon occur in other parts of the world, and by 2050 all regions except Africa will have nearly a quarter or more of their populations ≥60 [1]. Population ageing is projected to have a profound effect on societies by increasing demand on fiscal, political, healthcare, and social protection systems of many countries in coming decades. China will be one of the countries affected.

Healthcare associated-infections (HAIs) occur in patients under medical care in hospitals or other healthcare facilities. These infections occur during healthcare delivery for other diseases and even after discharge. HAIs are associated with prolonged hospital stay, poor prognosis, and increased mortality and economic burden [2–4]. A systematic review and meta-analysis reported that the major risk factors independently associated with HAIs were diabetes mellitus, immunosuppression, body temperature, surgery time in minutes, reoperation, cephalosporin exposure, days of exposure to central venous catheter, intensive care unit (ICU) admission, ICU stay in days, and mechanical ventilation [5]. Moreover, invasive devices such as catheters, ventilators, and central lines are associated with HAIs. The elderly are particularly vulnerable to infections due to reduced immunological competence and a high risk of underlying chronic illness [6–10]. However, there are limited data available regarding HAIs in elderly inpatients. Here we describe and analyze the results of a 3-year real-time surveillance study on the incidence of HAIs and the epidemiological and clinical characteristics of elderly inpatients in a large tertiary hospital in China.

## Methods

A cohort study based on real-time surveillance was performed from January 1, 2015 to December 31, 2017 in a large tertiary hospital with 1147 beds in Beijing, China. We carried out real-time surveillance of HAIs with an online nosocomial infection surveillance system (NISS) that can download inpatients’ clinical information and screen potential HAIs at any time and run automatically at an appointed time every day. Pediatric patients ≤14 years and patients who had been hospitalized for < 2 days or > 60 days were excluded.

All HAIs that occurred during hospitalization were identified by infection control practitioners and doctors. HAIs were defined as infections that occurred 48 h after the patients were admitted to hospital according to the 2001 definitions published by Ministry of Health of the People’s Republic of China [11]. We implement post-discharge surveillance of surgical site infection (SSI) for patients who undergo surgery. SSI included infection that occurred within 30 days after the procedure if no implant is left in place or within 1 year if an implant is placed.

The data were collected using an automatic online real-time NISS (RT-NISS, VERSION:12.8.2.1). The RT-NISS downloads and records clinical information for each participant including demographic characteristics, hospitalization days, diagnoses, operations, specific device days, body temperature, laboratory and auxiliary examinations, microbiologic profile, and bacterial resistance automatically online from other information systems including the hospital information system, electronic medical record, laboratory information system, picture archiving and communication system, mobile nursing information system, and anesthesia operation system. Then the RT-NISS automatically screens potential HAIs according to the 2001 definitions published by the Ministry of Public Health, which are input into the system by the algorithm of fever history, microbiological reports, serological and molecular testing, radiology information, and antibiotic usage [12].

The infection control team checked the collected data and removed invalid inputs. Then the infection control practitioners and doctors identified potential HAIs screened by RT-NISS according to the 2001 definitions published by Ministry of Health of the People’s Republic of China. This ensured that the collected data were valid and reliable. Previous studies showed that compared with a manual survey of HAIs (the gold standard) in all inpatients, the sensitivity and specificity of RT-NISS were 98.8 and 93.0%, respectively [12].

### Data analysis

SPSS 17.0 (SPSS Inc., Chicago, IL, USA) and Stata 9.0 (StataCorp, College Station, TX, USA) were used for data analysis. Rate ratios (RRs), 95% confidence intervals (CIs), and *P* values were calculated to identify significant differences in incidence density. Differences in categorical variables were assessed using χ^2^ tests. Statistical testing was performed at the conventional 2-tailed α = 0.05.

## Results

### Study population

During the 3-year study period, a total of 134,637 admissions with a total of 1,196,912 hospital days were involved in the study, including 60,332 (44.8%) elderly inpatients ≥60 years with 572,485 hospital days. The median hospital stay length was 7 days in total and 8 days in elderly. The median ages overall and for elderly were 58 and 69 years, respectively.

### HAIs in elderly and non-elderly patients

There were 2712 HAI cases among the 134,637 included patients with 1,196,912 patient-days, and the total incidence and incidence density of HAI were 2.0% and 2.3 per 1000 patient-days, respectively. The incidence density of HAI in elderly patients was significantly higher than in non-elderly under 60 years old and increased with age (Table 1). There were 1580 HAIs in 60,332 elderly inpatients and 1132 HAIs in 74,305 non-elderly inpatients, with a significantly higher incidence in elderly compared to non-elderly (2.6% vs. 1.5%, *χ*^*2*^ = 202.421, *P* < 0.001). The incidence of HAI in elderly inpatients significantly increased with age, with rates of 2.0% (637/32,156), 2.5% (462/18,203), and 4.8% (481/9973), respectively in patients aged 60–69 years, 70–79 years and ≥ 80 years (*χ*^*2*^ = 225.960, *P* < 0.001). The four departments with the highest HAI incidence rates were hematology and gerontology in the medicine department and the vascular and general surgical departments: 10.8, 6.5, 4.3, and 3.2% respectively. The departments with the lowest HAI incidence were otorhinolaryngology, gynecology and obstetrics, pain, and ophthalmology with 0.9, 0.5, 0.4, and 0.03%, respectively.

**Table 1:** Comparison of HAI incidence density in elderly and non-elderly patients

	Patient days	No. of HAIs	Incidence density (per 1000 patient-days)	RR	95% CI	*P*
Elderly (≥60 y)	572,485	1580	2.8	1.530	1.422–1.651	< 0.001
Non-elderly (< 60 y)	624,427	1126	1.8			

*CI* confidence interval, *RR* rate ratio

The ventilator and central line use rates in elderly patients were significantly higher than in non-elderly patients, but the urinary catheter use rate was lower in elderly patients. The incidence of ventilator-associated pneumonia (VAP) in elderly patients was lower than in non-elderly patients, catheter-associated urinary tract infections (CAUTIs) were significantly more common in elderly patients, and central line-associated bloodstream infection (CLABSI) rates were similar in both groups (Tables 2 and 3).

**Table 2:** Comparison of the device use rates in elderly and non-elderly patients

Device	Elderly (≥60 years) *N* = 60,332		Non-elderly (< 60 years) *N* = 74,305		*χ*^*2*^	*P*
	No. of use	Use rate%	No. of use	Use rate%		
Ventilator	1854	3.1	1544	2.1	134.015	< 0.001
Central line	3994	6.6	4103	5.5	71.046	< 0.001
Urinary catheter	16,376	27.1	20,577	27.7	5.048	0.025

**Table 3:** Comparison of the incidence densities of HAIs in elderly and non-elderly patients

Variable	Elderly (≥60 years)			Non-elderly (< 60 years)			RR	95% CI	*P*
	No. of cases	Ventilator/central line/urinary catheter days	Incidence density (per 1000 days)	No. of cases	Ventilator/central line/urinary catheter days	Incidence density (per 1000 days)			
VAP	57	15,379	3.7	76	9899	7.7	0.483	0.345–0.676	0.001
CLABSI	90	39,372	2.3	75	32,561	2.3	0.992	0.730–1.348	0.959
CAUTI	170	81,760	2.1	100	62,335	1.6	1.296	1.013–1.658	0.038

*CAUTI* catheter-associated urinary tract infection, *CLABSI* central line-associated bloodstream infection, *CI* confidence interval, *RR* rate ratio, *VAP* ventilator-associated pneumonia

### The main sites of HAIs in elderly patients

The top three sites of HAIs in elderly were the lower respiratory tract, urinary system, and bloodstream. Among these three sites, the rates of VAP, CAUTI, and CLABSI were 7.1% (57/802), 61.4% (170/277), and 33.3% (90/270), respectively (Table 4).

**Table 4:** HAI sites in the elderly

HAIs site	Infection cases	Percentage %
Lower respiratory tract infections	802	42.7
Pneumonia (non-VAP)	571	30.4
Tracheobronchitis	174	9.3
VAP	57	3.0
Urinary system infections	277	14.7
Non-CAUTI	107	5.7
CAUTI	170	9.0
Blood stream infections	270	14.4
Non-CLABSI	180	9.6
CLABSI	90	4.8
Digestive system infections	189	10.1
Antibiotic-associated diarrhea	121	6.4
Others	68	3.6
Surgical site infections	68	3.6
Skin and soft tissue infections	49	2.6
Upper respiratory tract infections	40	2.1
Oral infections	34	1.8
Central nervous system infections	31	1.7
Other infections	120	6.4
Total	1880	100.0

*CAUTI* catheter-associated urinary tract infection, *CLABSI* central line-associated bloodstream infection, *HAIs* healthcare associated-infections, *VAP* ventilator-associated pneumonia

### Comorbidities and special medical procedures

The numbers of male patients and patients with comorbidities and special medical procedures (e.g., neurological and chronic noncommunicable diseases and ICU admission) were significantly higher in the elderly, but fewer patients in that group underwent surgery (Table 5).

**Table 5:** Comparison of the comorbidities and medical procedures in elderly and non-elderly

Variables	Elderly (≥60 years) N = 60,332		Non-elderly (< 60 years) N = 74,305		*χ*^*2*^	*P*
	No. of cases	%	No. of cases	%		
Sex						
Male	33,280	55.2	34,293	46.2	1081.172	< 0.001
Female	27,052	44.8	40,012	53.8		
ICU admission	8352	13.8	7507	10.1	448.296	< 0.001
Operation	27,705	45.9	36,762	49.5	168.488	< 0.001
Cerebral hemorrhage	2770	4.6	3113	4.2	12.863	< 0.001
Cerebral infarction	19,779	32.8	11,350	15.3	5742.483	< 0.001
Brain neoplasms	444	0.7	269	0.4	88.371	< 0.001
Hypertension	34,050	56.4	17,931	24.1	14,661.554	< 0.001
Hyperlipidemia	8453	14.0	7424	10.0	517.187	< 0.001
Diabetes mellitus	18,885	31.3	11,827	15.6	4476.053	< 0.001
Coronary artery disease	18,352	30.4	5871	7.9	11,442.063	< 0.001
COPD	2126	3.5	302	0.4	1827.284	< 0.001
Malignant tumor	10,409	17.3	8011	10.8	1180.859	< 0.001
Malignant hematonosis	2513	4.2	1922	2.6	260.489	< 0.001
Osteoarthropathy	3207	5.3	2523	3.4	301.271	< 0.001

*COPD* chronic obstructive pulmonary disease, *ICU* intensive care unit

### The main pathogens detected in elderly HAI patients

The top five pathogens detected in elderly HAI patients were *Candida albicans*, *Klebsiella pneumonia*, *Acinetobacter baumannii*, *Escherichia coli*, and *Pseudomonas aeruginosa*. However, the main pathogens differed by infection site (Table 6).

**Table 6:** Most common pathogens detected in elderly HAI patients

HAI site	Pathogen	Strain (percentage %)
Lower respiratory tract	*Acinetobacter baumannii*	90(18.0)
	*Candida albicans*	88 (17.6)
	*Klebsiella pneumoniae*	71(14.2)
	*Pseudomonas aeruginosa*	55(11.0)
	*Stenotrophomonas maltophilia*	37(7.4)
	Others	158(31.7)
	Subtotal	499(100.0)
Urinary system	*Candida albicans*	55(24.4)
	*Escherichia coli*	39(17.3)
	*Enterococcus faecium*	36(16.0)
	*Candida tropicalis*	20(8.9)
	*Klebsiella pneumoniae*	17(7.6)
	Others	58(25.8)
	Subtotal	225(100.0)
Blood stream	*Escherichia coli*	39(13.2)
	*Klebsiella pneumoniae*	32(10.9)
	*Staphylococcus epidermidis*	20(6.8)
	*Enterococcus faecium*	20(6.8)
	*Staphylococcus hominis*	17(5.8)
	*Staphylococcus aureus*	12(4.1)
	Others	155(52.5)
	Subtotal	295(100.0)
Other	*Escherichia coli*	13(12.2)
	*Klebsiella pneumoniae*	10(9.4)
	*Acinetobacter baumannii*	7(6.5)
	*Staphylococcus aureus*	7(6.5)
	*Pseudomonas aeruginosa*	6(5.6)
	Others	64(59.8)
	Subtotal	107(100.0)
Total	*Candida albicans*	166(14.7)
	*Klebsiella pneumoniae*	130(11.6)
	*Acinetobacter baumannii*	121(10.8)
	*Escherichia coli*	108(9.6)
	*Pseudomonas aeruginosa*	87(7.7)
	Others	514(45.7)
	Subtotal	1126(100.0)

*HAI* healthcare-associated infection

## Discussion

Analysis of 3 years of data showed that the incidence and incidence density of HAIs in elderly patients were significantly higher than in non-elderly patients (2.6% vs. 1.5%, 2.8/1000 d vs. 1.8/1000 d, *P* < 0.05). The incidence of HAIs in the oldest group (≥80 years) was three-fold higher than that in non-elderly (4.8% vs. 1.5%, *P* < 0.05). Although the incidence of HAIs in this study was lower than most previous publications, the trend was consistent. HAIs accounts for 3.5–9% of all infections in developed and developing countries [13]. Extensive studies in the USA and Europe showed that HAI incidence density ranged from 13.0 to 20.3 episodes per 1000 patient-days [14]. Incidence rates are higher in ICUs, affecting ~ 30% of patients [15]. However, a survey of long-term care facilities for the elderly in Japan found that the overall incidence rate of HAIs was 0.18 per 1000 resident-days [16]. A survey of the prevalence of HAIs in older people in acute care hospitals in Scotland found a linear relationship between HAI prevalence and increasing age, the incidence of HAIs in patients younger and older than 65 were 7.37 and 11.13%, respectively [13]. An investigation of the HAI incidence in elderly hospitalized patients at a hospital in Hunan Province, China reported that the HAI incidence in patients aged ≥65 was significantly higher than in those aged < 65 (3.53% vs. 0.98%, χ^2^ = 354.44, *P* < 0.001) [17]. We may have underestimated the HAI incidence because we did not implement post-discharge surveillance for all inpatients. The lower HAI incidence may also be related to the shorter average hospital stay. In this study, the median lengths were only 7 days overall and 8 days for elderly patients.

Elderly inpatients are at high risk of HAI because of their poor immune function, decreased mobility, and comorbid chronic noncommunicable diseases such as cardiovascular disease, cancer, diabetes, and chronic obstructive pulmonary disease (COPD). Furthermore, elderly inpatients with HAIs have poor prognosis and increased economic burden [18]. One study evaluated elderly patients who had an HAI in the ICU and found that clinical outcomes of the elderly who acquired an infection in the ICU were influenced by sociodemographic and clinical variables that increase mortality rates [19]. Another study of HAI in elderly patients identified the following risk factors: advanced age; comorbid neurological and chronic noncommunicable diseases such as cerebral hemorrhage, cerebral infarction, brain neoplasms, diabetes mellitus, coronary artery disease, malignant tumor and malignant hematonosis; hospital days before HAI; ICU admission; and device use [9]. The participants of that study were elderly ≥60 years with or without HAIs. To control for confounders and identify novel risk factors of HAI in this population, we investigated comorbidities and special medical procedures in elderly and non-elderly groups. The percentages of male patients, patients with comorbidities (e.g., cerebrovascular disease, brain neoplasms, hypertension, hyperlipidemia, diabetes mellitus, coronary artery disease, COPD, malignant tumor, malignant hematonosis, and osteoarthropathy), and ICU admissions were significantly higher in the elderly group. Conversely, fewer elderly patients underwent surgery lower. These results suggest that the higher incidence of HAI in elderly may be attributable to the higher rates of comorbidities and special medical procedures in elderly inpatients.

One of the primary concerns of the current investigation was identifying common HAIs sites in elderly inpatients. Most HAIs were found in the lower respiratory tract, urinary system, and bloodstream. These findings are consistent with other available studies of both elderly and non-elderly inpatients [11, 20]. With recent improvements in implants, it is important to focus on device-associated infections (DAIs). The International Nosocomial Infection Control Consortium (INICC) and National Healthcare Safety Network reported monitoring data of HAIs in general and DAIs in particular. The INICC recorded the data of 861,284 patients hospitalized in INICC hospital ICUs in 50 countries for an aggregate of 3,506,562 days from 2010 to 2015. There were 6.2–19.2 cases of VAP per 1000 mechanical ventilator-days, 1.44–10.14 CLABSIs per 1000 central line-days, and 1.66–17.17 CAUTIs per 1000 catheter-days [21].

For elderly inpatients with lower respiratory tract, urinary system, and bloodstream infections, the percentages of VAP, CAUTI, and CLABSI were 7.1% (57/802), 61.4% (170/277), and 33.3% (90/270), respectively. Ventilator and central line use rates in the elderly group were significantly higher than in the non-elderly, but the elderly had a lower urinary catheter use rate. The incidence density of VAP in elderly was lower than in non-elderly, but CAUTIs were significantly higher, and CLABSI rates were similar. These results suggest that the incidence densities of VAP, CLABSI, and CAUTI in elderly inpatients did not increase with the ventilator, central line, and urinary catheter use rates. The lower incidence density of VAP in the elderly group was probably because the admitting diagnosis often included lower respiratory tract infections, and it was difficult to find evidence of VAP in these patients. The high incidence density of CAUTI in elderly inpatients is consistent with other reports. A study concerned with risk factors for CAUTI in Italian elderly found that increasing age and duration of hospital stay before catheter insertion were associated with CAUTIs [22]. The high percentage of CAUTI may be related to specific issues of elderly inpatients, but also due to failures in catheter insertion as we observed here and which may be our next study. The low similar incidence density of CLABSI in both the elderly and non-elderly groups may be related to effective interventions to prevent CLABSI. Hallam and colleagues collected data over a 5-year period and found a significant and sustained reduction in the CLABSI rate from 5 per 1000 catheter days to 0.23 per 1000 catheter days [23].

The other notable finding of the current investigation was the pathogens detected in elderly HAI patients. The five most common were *Candida albicans*, *Klebsiella pneumonia*, *Acinetobacter baumannii*, *Escherichia coli*, and *Pseudomonas aeruginosa*, but they varied by infection site. Extensive use of broad-spectrum antibiotics in elderly could account for the high positive detection rate of *Candida albicans*. The main pathogens of lower respiratory tract, urinary system, and bloodstream infections detected in elderly HAI patients could serve as reference evidence for empirical use of antibiotics to treat HAIs.

### Study limitations

First, this was a single-center study, so our findings cannot be generalized to all elderly patients in China. Second, we may have underestimated the HAI incidence because we did not implement post-discharge surveillance for all inpatients. Finally, the microorganism profile did not include drug sensitivity or antibiotic resistance. We will include those tests in our next study to better prevent HAIs in elderly inpatients.

## Conclusions

We observed a significantly higher overall incidence of HAIs for inpatients ≥60 years old compared to those under 60, which was attributable to higher rates of comorbidities, special medical procedures to treat neurological and chronic noncommunicable diseases, ICU admission, and surgery in elderly. The main sites of HAIs in elderly patients were the lower respiratory tract, urinary system, and bloodstream due to high rates of VAP, CLABSI and CAUTI. Interestingly, the incidence density of device-associated infections did not increase with the higher device use rate in the elderly group. Futures studies to identify risks factors of HAIs in elderly will help decrease the rate in elderly inpatients.

## Data Availability

The datasets generated and/or analyzed during the current study are not publicly available due the data copyright protection of the author’s institute, but are available from the corresponding author on reasonable request.

## Abbreviations

CAUTI: Catheter-associated urinary tract infection
CI: Confidence interval
CLABSI: Central line-associated bloodstream infections
HAI: Healthcare-associated infections
ICU: Intensive care unit
NISS: Nosocomial infection surveillance system
VAP: Ventilator-associated pneumonia

## References

[1] World population prospects: the 2017 revision, key findings and advance tables. https://reliefweb.int/report/world/world-population-prospects-2017-revision-key-findings-and-advance-tables. Accessed 05 Mar 2018.

[2] Kritsotakis EI, Kontopidou F, Astrinaki E, Roumbelaki M, Ioannidou E, Gikas A (2017). Prevalence, incidence burden, and clinical impact of healthcare-associated infections and antimicrobial resistance: a national prevalent cohort study in acute care hospitals in Greece. Infect Drug Resist.

[3] Graves N, Weinhold D, Tong E, Birrell F, Doidge S, Ramritu P (2007). Effect of healthcare-acquired infection on length of hospital stay and cost. Infect Control Hosp Epidemiol.

[4] Vrijens F, Hulstaert F, Devriese S, van de Sande S (2012). Hospital-acquired infections in Belgian acute-care hospitals: an estimation of their global impact on mortality, length of stay and healthcare costs. Epidemiol Infect.

[5] Rodríguez-Acelas AL, de Abreu AM, Engelman B, Cañon-Montañez W (2017). Risk factors for health care–associated infection in hospitalized adults: systematic review and meta-analysis. Am J Infect Control.

[6] Llopis F, Ferré C, García-Lamberechts EJ, Martínez-Ortiz-de-Zárate M, Jacob J, González-Del-Castillo J (2016). Are short-stay units an appropriate resource for hospitalising elderly patients with infection?. Rev Calid Asist.

[7] Kemp M, Holt H, Holm A, Kolmos HJ (2013). Elderly patients are at high risk from hospital-acquired infection. Ugeskr Laeger.

[8] Yahav D, Eliakim-Raz N, Leibovici L, Paul M (2016). Bloodstream infections in older patients. Virulence..

[9] Zhao X, Wang L, Wei N, Zhang J, Ma W, Zhao H (2019). Risk factors of health care–associated infection in elderly patients: a retrospective cohort study performed at a tertiary Hospital in China. BMC Geriatr.

[10] Castle SC (2000). Clinical relevance of age-related immune dysfunction. Clin Infect Dis.

[11] The Ministry of Public Health (2001). The nosocomial infections diagnosis criterion. Natl Med J China.

[12] Du M, Xing Y, Suo J, Liu B, Jia N, Huo R (2014). Real-time automatic hospital-wide surveillance of nosocomial infections and outbreaks in a large Chinese tertiary hospital. BMC Med Inform Decis Mak.

[13] Cairns S, Reilly J, Stewart S, Tolson D, Godwin J, Knight P (2011). The prevalence of health care-associated infection in older people in acute care hospitals. Infect Control Hosp Epidemiol.

[14] Khan HA, Baig FK, Mehboob R (2017). Nosocomial infections: epidemiology, prevention, control and surveillance. Asian Pac J Trop Biomed.

[15] Abulhasan YB, Rachel SP, Châtillon-Angle MO, Alabdulraheem N, Schiller I, Dendukuri N (2018). Healthcare-associated infections in the neurological intensive care unit: results of a 6-year surveillance study at a major tertiary care center. Am J Infect Control.

[16] Kariya N, Sakon N, Komano J, Tomono K, Iso H (2018). Current prevention and control of health care-associated infections in long-term care facilities for the elderly in Japan. J Infect Chemother.

[17] Wang J-J, Fan S-Y, Zhang N (2015). Incidence of healthcare-associated infection in elderly hospitalized patients at a hospital in Hunan Province. Chin J Infect Control.

[18] Kurutkan MN, Kara O, Eraslan İH (2015). An implementation on the social cost of hospital acquired infections. Int J Clin Exp Med.

[19] Sousa ÁFL, Queiroz AAFLN, Oliveira LB, Moura LKB, Andrade D, Watanabe E (2017). Deaths among the elderly with ICU infections. Rev Bras Enferm.

[20] Ali HS, Khan FY, George S, Shaikh N, Al-Ajmi J (2016). Epidemiology and outcome of ventilator-associated pneumonia in a heterogeneous ICU population in Qatar. Biomed Res Int.

[21] Rosenthal VD, Al-Abdely HM, El-Kholy AA, AlKhawaja SAA, Leblebicioglu H, Mehta Y (2016). International nosocomial infection control consortium report, data summary of 50 countries for 2010-2015: device-associated module. Am J Infect Control.

[22] Vincitorio D, Barbadoro P, Pennacchietti L, Pellegrini I, David S, Ponzio E (2014). Risk factors for catheter-associated urinary tract infection in Italian elderly. Am J Infect Control.

[23] Hallam C, Jackson T, Rajgopal A, Russell B (2018). Establishing catheter-related bloodstream infection surveillance to drive improvement. J Infect Prev.

